# Design, implementation, and operation of a rapid, robust named entity recognition web service

**DOI:** 10.1186/s13321-019-0344-9

**Published:** 2019-03-08

**Authors:** Sune Pletscher-Frankild, Lars Juhl Jensen

**Affiliations:** 1 Disease Systems Biology Program, Novo Nordisk Foundation Center for Protein Research, Faculty of Health and Medical Sciences, University of Copenhagen, Copenhagen, Denmark; 2 Present Address: Intomics A/S, Lyngby, Denmark

**Keywords:** Text mining, Named entity recognition, Web services

## Abstract

Most BioCreative tasks to date have focused on assessing the quality of text-mining annotations in terms of precision and recall. Interoperability, speed, and stability are, however, other important factors to consider for practical applications of text mining. For about a decade, we have run named entity recognition (NER) web services, which are designed to be efficient, implemented using a multi-threaded queueing system to robustly handle many simultaneous requests, and hosted at a supercomputer facility. To participate in this new task, we extended the existing NER tagging service with support for the BeCalm API. The tagger suffered no downtime during the challenge and, as in earlier tests, proved to be highly efficient, consistently processing requests of 5000 abstracts in less than half a minute. In fact, the majority of this time was spent not on the NER task but rather on retrieving the document texts from the challenge servers. The latter was found to be the main bottleneck even when hosting a copy of the tagging service on a Raspberry Pi 3, showing that local document storage or caching would be desirable features to include in future revisions of the API standard.

**Electronic supplementary material:**

The online version of this article (10.1186/s13321-019-0344-9) contains supplementary material, which is available to authorized users.

## Introduction

BioCreative and other shared tasks in the biomedical text-mining community have over the years played a key role in progressively improving text mining methods, in particular for named entity recognition (NER). Most BioCreative tasks have focused purely on evaluating the precision and recall [1, 2], with the BioC interoperability task [3] and the interactive annotation task (IAT) [4] being notable exceptions. However, as illustrated by the latter two tasks, whereas precision and recall are obviously important factors, they are far from the only factors that matter when using text mining in practice. Interoperability, speed, and stability are other very important factors; the new Technical Interoperability and Performance of annotation Servers (TIPS) task set out to evaluate just that.

Running fast and robust web services is not trivial. Many academic online tools will become unresponsive or even crash if subjected to many simultaneous requests, e.g. when using them on a practical course. Also, it is impossible to ensure near perfect uptime unless the services are hosted professionally with reliable power and internet connection. Handling these issues requires a focus on the engineering aspects rather than only on the scientific quality of the tools.

We participated in the BioCreative V IAT [4] with the interactive annotation tool, EXTRACT, which helps curators find and extract standard-compliant terms for annotation of metagenomic records and other samples [5]. Behind its web-based user interface, the system makes use of the same real-time tagger for NER as the augmented browsing tool Reflect [6]. The core NER engine was designed from the ground up with speed in mind and is capable of tagging thousands of PubMed abstracts per second per CPU core [7]. This makes it ideally suited for large-scale and real-time applications, such as the TIPS task.

Here, we present the entire system for the NER web service that underlies the EXTRACT [5] and Reflect tools [6] as well as our entry in the TIPS challenge. This includes not only the tagger software itself, which has been described in several earlier publications, but also the Mamba web server software that enables us to host multi-threaded web services with preloaded data (e.g. the tagger dictionary) and priority queues to efficiently handle even high request rates. The system delivered a total turn-around time of about 1 s for small requests, and was able to process approximately 5000–10,000 abstracts per minutes for larger batch requests. Notably, the vast majority of this time was spent on retrieving the document text rather than actual processing of it; to make the server faster, it would thus be necessary to locally store or cache the documents, which was explicitly not permitted in the TIPS task.

## Methods

### Dictionaries used for NER and normalization

The server uses a combination of previously published dictionaries to recognize six of the types of entities accepted by the BeCalm server and normalize them to identifiers from databases and ontologies. These are a subset of the entity types used in EXTRACT v2 [5].

For annotation of gene/protein names, the tagger uses a dictionary covering the 9.6 million protein-coding genes from 2031 organisms included in STRING v10.5 [8] as well as ncRNAs from the RAIN database [9]. Unlike many NER systems, the BeCalm API makes a distinction between genes and their protein products. Because the STRING database is locus-based, i.e. it does not distinguish between splice isoforms, and because ncRNAs are also included, we chose to use the type *GENE* for these annotations and to not support the *PROTEIN* annotation type. All recognized names are disambiguated to their respective STRING or RAIN identifiers, which are derived from the Ensembl [10], RefSeq [11], and miRBase [12] databases.

Annotations of the type *CHEMICAL* are made using a dictionary comprised of small-molecule compounds from the PubChem database [13], which was developed and used for recognition of chemical names in STITCH v5 [14]. All annotations of chemicals are normalized to PubChem compound identifiers.

The tagger makes annotations of the type *ORGANISM* using an updated version of the dictionary of the SPECIES/ORGANISMS tagger [7]. The dictionary was constructed based on NCBI Taxonomy [10], and all annotations are thus normalized to NCBI taxon identifiers.

To perform annotations of the types *SUBCELLULAR_STRUCTURE*, *TISSUE_AND_ORGAN*, and *DISEASE* the tagger uses the dictionaries created as part of the COMPARTMENTS [15], TISSUES [16, 17], and DISEASES [18] database, respectively. These were constructed from Gene Ontology [19], Brenda Tissue Ontology [20], and Disease Ontology [21], identifiers from which are used for normalization of the annotations.

The version of the complete dictionary used by Tagger for the TIPS task has been deposited on FigShare (10.6084/m9.figshare.4578292). The reduced dictionary used by PiTagger has also been deposited on Figshare (10.6084/m9.figshare.4635175). The latest version of the dictionary, which is used by the production server, is available at http://download.jensenlab.org/tagger_dictionary.tar.gz.

### Named entity recognition software

The core of the NER system is a highly optimized dictionary-based tagger engine, implemented in C++. It is able to perform flexible matching of a dictionary with millions of names against thousands of abstracts per second per CPU core [7]. The tagger is furthermore inherently thread safe, for which reason a single instance of the tagger can easily handle many parallel requests. These properties make it an excellent starting point for building a real-time service that can handle large requests as required for TIPS task.

Although the TIPS task does not assess the quality of the annotations, it is worth noting that the speed of the tagger was not achieved by sacrificing quality. The quality of the tagging results for organism names was previously evaluated on gold-standard corpora and found to be comparable to the best methods with precision and recall of ~ 83% and ~ 73%, respectively [7, 22]. The NER quality has not been benchmarked directly for chemicals, genes, tissues, and diseases; however, these NER components have shown to give good results when used as the basis for association extraction [8, 9, 13, 15–18].

The tagger software is open source and available at https://bitbucket.org/larsjuhljensen/tagger/. It can be used either as a command-line tool or as a Python module. It is also distributed as a Docker container at https://hub.docker.com/r/larsjuhljensen/tagger/.

### Mamba web service framework

To be able to robustly host web services, we developed an in-house Python framework, Mamba, which can simultaneously run several compute-intensive requests in parallel while remaining highly responsive to small requests. The framework has a modular structure that enables us to use it both for the tagger, which is the focus of this paper, and for serving precomputed results from relational database through REST APIs and interactive web interfaces. The REST API code accesses a single instance of the tagger engine through its Python module, which preloads the complete dictionary into RAM when the Mamba server is started.

Mamba is a stand-alone multithreaded Python webserver created specifically to expose computationally heavy and/or heavily requested services as web services. Mamba was designed to solve two main objectives native to websites exposing a computational pipeline. Firstly, Mamba protects against clients overloading the backend system by controlling computational resources in terms number of simultaneous requests and memory usage, which is all specified in a standard configuration file format. Secondly, Mamba is designed to be as simple as possible to set up and run while allowing fine-grained resource control and providing a plugin-framework for project-specific code, such as the tagger. Plugins implement their specialized functionality as a Python API class per request type.

Mamba uses task queues combined with a configurable number of worker-threads handling the queued tasks. The length of the queue combined with the number of worker threads provide the first level of resource control, which prevents that too many tasks are executed at the same time, which could cause the service to crash, or that the queue becomes excessively long, which could make the service unavailable for extended periods of time. In addition to that, Mamba comes with configurable per user and overall resource restrictions, which limit the maximum number of simultaneous requests and the memory usage used per IP address. When these limits are reached, requests will be rejected using the appropriate HTTP status code.

Each Mamba server will require task specific parsing and processing of input data, and Mamba provides a task-based API for this that is centered around a single virtual Mamba request class. The configuration file specifies where Mamba should look for project-specific Request classes, which will be loaded as Python code files when starting the server, also allowing data such as the dictionary to be preloaded. The API allows tasks to jump between task queues, which represents different stages of the task. For example, we use separate queues for the tagger to do the initial parsing of the request, to download the document text from the document server, and to perform the actual named entity recognition. This enables us to specify that many document download tasks can take place in parallel (which is not a CPU intensive), but that only a few actual NER tagging tasks can run in parallel (the CPU intensive part of a request). When configured appropriately, this makes the Mamba server very responsive to all users as it is constantly aware of preventing system from overloading.

To further improve the response time, stability, and handling of multiple requests, even in the thousands, the main input mechanism is controlled by a single POSIX select statement. Mamba listens to a single port through a select statement that runs as single main thread; this sets it apart from most other Python-based web-service frameworks, which handle each input as new thread. The select statement collects data for all incoming requests as they are received over the network and adds a new task to the parse queue only once a full request has been received. From thereon the processing is handled by the thread pools described above, until all processing has been done and the result is ready to be sent back to the client. Mamba then uses the same efficient select statement that receives the incoming data to send the result back as an HTTP response to the client. Mamba automatically cleans up incomplete requests caused by communication errors, restarts worker threads if they crash, and catches any exceptions produced by Mamba or its plugins, returning the appropriate HTTP status code.

Implementing the BeCalm API itself simply involved adding a request class the Mamba tagger module. Different parts of the BeCalm API run in different queues to ensure that e.g. *getStatus* requests and the initial parsing of *getAnnotations* requests are executed immediately, that many document downloads can run in parallel, and that not too many of the actual CPU-intensive NER tasks are running at the same time. The queues used for this are not exclusive to BeCalm API, but are shared with the other tagger APIs and the EXTRACT tool [4].

### Hardware and hosting

The main tagger runs on a single server with one Intel Xeon E5-2620 2.4 GHz CPU and 256 GB of RAM. This server also runs many other resources and databases related to text mining, including EXTRACT [5], SPECIES/ORGANISMS [7], COMPARTMENTS [15], TISSUES [16, 17], and DISEASES [18]. This server—from hereon referred to as Tagger—is hosted at the high-performance computing facility Computerome (https://computerome.dtu.dk) that provides it with a highly reliable gigabit internet connection.

To test the influence of the performance of actual document tagging vs. overhead associated with fetching of document texts, we ran a second instance of the tagger software on a Raspberry Pi 3 with a 1.2 GHz quad-core ARM Cortex-A53 and 1 GB of RAM. Due to the limited memory, this instance runs with a reduced dictionary; however, it should be noted that tagging speed is largely independent of dictionary size because the tagging algorithm is based on hash lookups [7]. This instance was hosted over my home internet connection (60 Mbit/s download, 25 Mbit/s upload) and is in the following referred to as PiTagger.

## Results and discussion

### Rapid annotation of biomedical entities

To test the speed of Tagger and PiTagger when accessed through the BeCalm API, we submitted private requests for tagging of 1, 10, 100, 1000, and 5000 abstracts from the abstract and patent servers via the BeCalm web interface. All settings except from the number of documents to tag were left at their default values. Each of the five sizes of tagging requests was repeated five times at four different timepoints, giving a total of 20 observations of the total time required for tagging for each size of request from each document source on each of the two tagger servers. These results are summarized as means and standard deviations in Table 1; the detailed data with each individual observation are available in Additional file 1: Tables S1 and S2.

**Table 1:** Performance of the taggers

# Documents	Tagger: total time (s)		PiTagger: total time (s)	
	Abstract server	Patent server	Abstract server	Patent server
1	0.87 ± 0.32	0.84 ± 0.32	0.75 ± 0.34	0.84 ± 0.24
10	0.98 ± 0.30	0.83 ± 0.26	1.10 ± 0.28	0.87 ± 0.37
100	1.89 ± 0.29	1.48 ± 0.27	2.34 ± 0.33	1.52 ± 0.34
1000	11.31 ± 0.68	6.02 ± 0.37	15.23 ± 0.48	8.89 ± 0.48
5000	52.18 ± 2.76	26.67 ± 1.16	72.73 ± 1.81	40.83 ± 1.01

For small requests the total turnaround time is ~ 1 s. Larger requests take an extra 5–10 s per 1000 abstracts to be processed on Tagger. Notably, most of this time is spent on retrieving the document texts from document identifiers, whereas the actual NER step takes only about 20% of the total time. This is reflected in the fact that the PiTagger, which runs on a Raspberry Pi 3, takes only about 50% longer to process large requests

Neither Tagger nor PiTagger suffered any errors or slowdowns during these tests, despite the Tagger server hosting multiple other resources and the PiTagger running on minimal hardware. This shows that the software is not only fast but also stable. This is unsurprising since all parts except the BeCalm API-specific code have been used in a production setting for several years.

In summary, the Tagger speed tests showed that there is a constant overhead of about 1 s on all tagging requests, which dominates the picture up to tagging of about 100 patent abstracts. For larger requests, the service takes ~ 5 and ~ 10 s more per 1000 patent abstracts and PubMed abstracts, respectively. This difference is presumably explained by PubMed abstracts being, on average, about twice as long as patent abstracts. Notably, the vast majority of the time is spent on fetching the document texts, with only about ~ 20% of time being spent on actual processing. Although explicitly not permitted in the TIPS task, local storage or caching of documents on the annotation server would thus be an attractive future feature.

To further test and illustrate that retrieval of document texts is the main bottleneck, we set up a second copy of the tagger code, PiTagger, to run on a Raspberry Pi 3 with a reduced dictionary. However, as the tagging speed is largely independent of dictionary size, the performance numbers can nonetheless be directly compared. For small requests, the total time is indistinguishable between Tagger and PiTagger, and even for large requests PiTagger takes only about 50% longer than Tagger (Table 1). This is the case despite the service running only one thread per request, thus utilizes only a quarter of the compute power of a Raspberry Pi 3 in these tests. The PiTagger did not participate in the full official TIPS evaluation.

The total tagging time for the official TIPS requests was in the beginning consistently longer than for the private requests reported in Table 1, which were submitted during the same weeks. Monitoring the tagging services during TIPS requests revealed that actual document processing was as fast as always. In light of the results above, we assume that this slowdown was due to the fetching of documents taking longer in the official tests, when all participants simultaneously sent requests to the central document servers, thus making them even more of a bottleneck. It is thus also no surprise that the fast servers all performed equally well, since they all spent far more time on downloading the text documents and uploading the annotations than on processing them.

### Extending the BeCalm API

The BeCalm API in its current form has certain design constraints that limit the flexibility and thereby usefulness of the annotation servers. Firstly, document text is not submitted as part of the request, but must instead be fetched from designated sources based on the submitted document identifiers. Secondly, the results cannot be returned directly to the end user, but must be returned to the central BeCalm server. Through creative use of the *custom_parameters* part of the request, our implementation circumvents both of these constraints.

Instead of hardwiring the annotation server to use only the abstract and patent servers provided by BeCalm, the relationships between *source* and server URL are specified within a *servers* subsection of *custom_parameters*. This enables end users to obtain the tagging results for any desired documents, provided they make the documents available through an API compatible with the one used by the BeCalm document servers.

Similarly, the annotation server is not hardwired to return the annotation results to the BeCalm server. Instead, the *saveAnnotations* request will be made to the URL specified in as *apiurl* in the *custom_parameters* section. This allows end users to set up their own server to receive the results directly, if they so wish.

## Conclusions

Turning scientific software into a stable and fast web service can be a challenging engineering task. First, the software has to be sufficiently fast, which will often require optimization of the implementation. Second, it has to be made robust enough to handle many simultaneous requests without overloading the server. Third, it has to be hosted in a manner that ensures the server is available at all times.

In case of the Tagger NER software, the implementation itself was already highly optimized, since it was designed to be used on very large text corpora. The issues related to robustness of the web service were dealt with by the Mamba web service framework, which uses queues with associated thread pools to efficiently process multiple requests in parallel, while protecting the server against being overloaded if a user floods it with requests. Finally, we hosted the web service at a supercomputing facility to ensure high availability. The result was a server which indeed was among the fastest and suffered no downtime during the challenge.

However, even if a web service is both stable and fast, processing large amounts of text through web services can be inefficient. This is because transferring the input text and the output data to and from the web service can easily take much longer than the actual computations. When processing large text corpora, a better solution is thus usually to run the software locally. To make this as easy as possible, we make the Tagger software available as as a Docker container (https://hub.docker.com/r/larsjuhljensen/tagger/) and provide a download file with the dictionary used by the web service (http://download.jensenlab.org/tagger_dictionary.tar.gz).

## Additional file

**Additional file 1.** Supplementary tables with detailed performance evaluation data.
